# Autobiographical memory and episodic future thinking in anorexia nervosa: a three-year follow-up

**DOI:** 10.1186/s40337-025-01280-4

**Published:** 2025-05-30

**Authors:** Johanna Louise Keeler, Valentina Cardi, Georgia Peters-Gill, Hubertus Himmerich, Kate Tchanturia, Janet Treasure

**Affiliations:** 1 Department of Psychological Medicine, Centre for Research in Eating and Weight Disorders, Institute of Psychiatry, Psychology and Neuroscience, King’s College London, London, SE5 8AF UK; 2 Department of General Psychology, University of Padova, Padua, Italy; 3 The Advocacy People, Southampton, Hampshire UK; 4 South London and Maudsley NHS Foundation Trust, Bethlem Royal Hospital, Monks Orchard Road, Beckenham, Kent, BR3 3BX UK; 5 Illia State University, Tbilisi, Georgia

**Keywords:** Anorexia nervosa, Autobiographical memory, Eating disorders, Future thinking, Memory, Follow-up

## Abstract

**Background:**

People with anorexia nervosa (AN) show deficiencies with recalling specific details of autobiographical memories (AM). This may interfere with wider processes of self-narrative construction and identity building, which are a fundamental part of the recovery process. However, no studies have examined the temporal stability of AM deficiencies over time or their prognostic value in this population.

**Methods:**

This study followed up adults with mostly longstanding AN that participated in a previous study examining AM and episodic future thinking (EFT) abilities after 3 years. A total of 20 participants with AN responded (44% of the original sample) and repeated a remotely administered written version of the Autobiographical Memory Test (AMT) and Episodic Future Thinking Task (EFT-T) and a series of questionnaires. The word lists used were identical to the previous study, but were alternated for the present study (i.e., participants viewing list A for the AMT previously saw list B in the follow-up). Task outcomes included AM and EFT specificity, vividness, difficulty to remember/imagine, positivity, realisticness and detailedness.

**Results:**

Respondents had persistently high eating disorder psychopathology and comorbid psychiatric symptoms at follow-up. Body mass index (BMI) increased in most participants (n = 15), albeit the group average was 17.2 kg/m^2^. There was comparable performance on the AMT and EFT-T at both baseline and at follow-up. Analyses interrogating prognosis were not possible due to the homogeneity in ED-related outcomes, although baseline AM specificity was not related to BMI, ED symptoms, depressive symptoms or measures of identity functioning (i.e., consolidated identity, disturbed identity, or lack of identity), at follow-up.

**Conclusions:**

Problems with retrieving specific details of AMs show temporal stability over time in people with longstanding AN. The prognostic value of AM specificity on ED outcomes remains unknown, which future well-controlled prospective longitudinal studies could address.

**Supplementary Information:**

The online version contains supplementary material available at 10.1186/s40337-025-01280-4.

## Abstract (plain language summary)

People with anorexia nervosa (AN) often show difficulties with recalling events from their personal life and may show overgeneralisations in their memory recall, as well as biases in how they think about the future. It is unknown however whether these difficulties persist over time, get better when people recover, or perhaps even worsen when people remain unwell for long periods of time. In this study, people with AN that took part in a previous study looking at autobiographical memory recall and thinking about the future were followed up three years later, where they completed the same tasks. People that took part in this follow-up were largely unrecovered, meaning that it was not possible to examine whether memory abilities were related to poor or good outcomes over time, although memory abilities were not related to the follow-up BMI, eating disorder symptoms or depression symptoms. Participants still showed deficiencies in recalling specific details of personal memories at this time-point, suggesting that their memory may not worsen even with persisting illness.

**Supplementary Information:**

The online version contains supplementary material available at 10.1186/s40337-025-01280-4.

## Introduction

The course of anorexia nervosa (AN) is marked by severe undernutrition leading to low body weight, and a marked reduction in brain volume that is of the greatest magnitude of any psychiatric disorder [42]. There is consistent evidence for generalised (i.e., not cue-specific) problems with retrieving specific details of autobiographical memories (AM) in AN across all age categories [13], which may also be a transdiagnostic feature of many psychiatric disorders including mood disorders, post-traumatic stress disorder and psychosis [2]. Several theories have emphasised the importance of AM, particularly within early adulthood [32], for social functioning, the formation of a personal self-narrative, thinking about the future, and identity-building – a key aspect of recovery from AN [6, 8, 9]. However, the stability of this cognitive domain over time in this population has not been studied and whether AM abilities have prognostic value in AN is uncertain.

Longitudinal research over a decade has found that AM declines throughout life, even in individuals who are otherwise cognitively healthy [12]. Declining AM specificity may be an early indicator of other cognitive deficiencies [12]. In adolescents and young adults, overgeneral memory (OGM) as measured by the Autobiographical Memory Test (AMT), referring to a difficulty in retrieving specific AMs, has been found to be significantly but modestly stable over time (3–6 years) with a Spearman’s coefficient of 0.31 [37]. This was found regardless of whether the participant had a history of major depressive disorder or not. Another study in six independent samples examined test–retest reliability at shorter intervals ranging from 1 to 5 months, finding slightly larger correlation coefficients ranging from 0.53 to 0.68 [30].

There have been few longitudinal studies assessing changes, or stability, in OGM in people with AN. Two studies have explored changes in AM functioning over the course of treatment. In one study by Terhoeven et al. [39], 24 patients with AN were assessed over the course of inpatient treatment (mean duration 11 weeks). Akin to the wider literature, baseline poor performance on the AMT was found, which was not modified by cue type. At the end of treatment (average BMI increase of 3.1 kg/m^2^), it was found that AM specificity had improved for memories elicited by depression-related and neutral cues, but not for food- and body-related cues. A conflicting study by Tenconi et al. [38] assessed 56 patients with AN after 23 weeks of weight restoration treatment, finding no changes in AMT score after treatment, although they did not report results according to cue type. However, there was a trend towards an improvement in AM specificity of recent memories. No study has examined longer-term changes in AM or EFT abilities, although a prior cross-sectional study found that people long-term recovered from AN were similar to healthy controls in their ability to recall specific AMs and generate specific EFTs, despite also reporting more subjective difficulty in recalling memories [21, 22].

To our knowledge, no study has assessed the prognostic value of baseline AM functioning in relation to improvements in ED psychopathology, weight or other measures of common psychopathology (e.g., depression) in people with AN. In other populations, OGM (poor AM specificity) has been found to predict linear increases in depression over a period of 18 months in a community sample [41]. It has also been found to predict later symptoms of depression in a prospective adolescent sample [43] and in clinically-diagnosed populations with MDD [24]. Given the transdiagnostic similarities between mood disorders and AN (e.g., anhedonia; [4]) and the high comorbidity rate [40], it is conceivable that AM abilities may also be related to mood-related or eating disorder-related outcomes in people with AN. Moreover, as aforementioned OGM may interrupt the understanding of one’s personal narrative and thus identity formation and reconciliation [21, 22], which may also be related to outcomes in people with AN.

The aim of the present study was primarily to examine the temporal stability of AM and EFT performance. A three-year follow-up study was conducted using the sample from a prior study that examined AM and EFT abilities using the Autobiographical Memory Test and Episodic Future Thinking Task in a sample of adults with mostly longstanding AN [21, 22]. This study found evidence for AM deficiencies in AN in response to all cue types (positive, neutral and disorder-related), but not EFT deficiencies. In this exploratory follow-up study, we also aimed to investigate whether AM specificity was sensitive to outcome, i.e., remained poor in participants who remained unrecovered according to BMI and ED psychopathology, and improved in participants who recovered. As an exploratory aim we aimed to examine associations between baseline AM specificity and follow-up outcomes, including variables relating to identitfy functioning, in order to identify whether AMT task performance has prognostic value, and to inform future longitudinal studies.

## Methods

### Participants and design

This study was a follow-up of a previously published cross-sectional, between-groups study [21, 22]. A total of 121 participants took part in the original study: 46 with acute AN (acAN), 40 individuals recovered from AN (recAN), and 35 healthy controls (HC) with no current or previous psychiatric or neurological disorders. Participants were recruited from the South London and Maudsley NHS Trust, email circulars at King’s College London and social media.

At entry to the original study, the acAN group was required to have a current diagnosis of AN from a clinician, which was verbally reported during a pre-study phone call where a full clinical history was taken, and a body mass index ≤ 18.5 kg/m^2^. Participants also had to: be ≥ 18 years old; fluent in the English language; have no history of post-traumatic stress disorder, substance abuse or psychotic disorders; have access to a computer and a stable internet connection; have no uncorrected auditory or visual impairments; and have no aphantasia as determined by the Vividness of Visual Imagery Questionnaire. For full inclusion and exclusion criteria of the recAN and HC samples, see [21, 22].

For this study, participants that were originally in the acAN group and who had consented to being recontacted for future studies were approached via an email invitation. A total of 20 participants from the original acAN group (44%) participated in this follow-up study. The interval between baseline and follow-up was almost three years (M ± SD months = 33.2 ± 1.0, range 30.6–34.5 months). There were no observable differences in relevant baseline characteristics between those who responded to the advertisement and those who did not (Table S1).

### Measures

#### Demographic and clinical variables

Amongst other variables, the following were measured in a bespoke demographic questionnaire: age; gender; years of education; medication usage; living status; employment status.

#### Assessment of events in interim period

Life events occurring in the time period between baseline and follow-up were assessed using the Life Events Questionnaire, in order to identify any potential intercurrent events that may affect autobiographical memory abilities, such as traumatic experiences (LEQ; [29, 33]). The LEQ is an 82-item questionnaire where participants mark the life events or changes that have occurred within a defined period (in this study, the last three years). Participants then indicate whether the event was considered “good” or “bad” and its impact on a 4-point scale ranging from 0 (“No effect”) to 3 (“Great effect”). Three scores are obtained from the questionnaire as a sum of all the impact ratings: a negative events score, a positive events score, and a total events score.

Participants were also asked to report their usage of services for the treatment of their eating disorder within the last 3 years.

#### Eating disorder psychopathology and characteristics

The Eating Disorder Examination Questionnaire 6.0 (EDE-Q; [11]) was used to quantify eating disorder psychopathology over the past 28 days [3]. The Cronbach’s alpha at baseline was 0.89 and at follow-up was 0.84, and the Global score showed moderate test–retest reliability (*r* = 0.60; Table S2). Participants were also asked to self-report their current ED diagnosis, AN subtype (restricting, or binge-purge), self-perceived recovery status (and a definition of how they characterise this), current use of treatment for their EDs, and time in years since their ED diagnosis.

#### Psychiatric comorbidity and psychopathology

Symptoms of comorbid psychiatric symptoms were assessed using the Depression, Anxiety and Stress Scale-21 (DASS-21; [17, 25]) and the Generalised Anxiety Disorder Assessment (GAD-7; [19, 36]). The DASS-21 is a 21-item self-report questionnaire that assesses symptoms of depression, anxiety and stress over the previous week (Cronbach’s alpha at baseline = 0.95, at follow-up = 0.91; test–retest reliability *r* = 0.38–0.74; Table S2). The GAD-7 is a 7-item scale assessing symptoms of generalised anxiety disorder over the previous two weeks (alpha at baseline = 0.91, at follow-up = 0.90; test–retest reliability *r* = 0.48; Table S2). Participants were also asked to self-report their diagnoses of comorbid psychiatric disorders.

#### Identity and self-concept

The Self Concept and Identity Measure (SCIM; [20]) is a 27-item self-report measure developed to assess identity consolidation and normative and problematic dimensions of identity functioning. The measure results in three subscales: (1) the consolidated identity scale, capturing a sense of knowing oneself, consistency in beliefs and values, and positive self worth; (2) the disturbed identity scale, assessing discontinuity in a person’s opinions, beliefs and/or values, and overdependence on others for understanding or defining one’s identity, and (3) the lack of identity scale, which captures feelings of being lost, broken, or empty, or not knowing who oneself is. The Cronbach’s alpha for this study was 0.86.

#### Autobiographical memory and future thinking

To assess the specificity of autobiographical memories and simulated future episodes, an online written version of the Autobiographical Memory Test (AMT; [44]) and a future-thinking variant of the AMT (the EFT task [EFT-T]; [16]) was used. The AMT and EFT have both shown good construct and convergent validity [15]. We calculated the Cronbach’s alpha using the coding of the primary outcome (specificity) of the task. The alpha for the AMT was 0.64 at baseline and 0.77 at follow-up; the alpha for the EFT-T was 0.67 at baseline and 0.73 at follow-up. The test–retest reliability after the 3-year interval was generally low across outcomes in this sample (Table S2). Whilst no studies have investigated the psychometric properties of the AMT/EFT-T when delivered online, a previous study found that participants performed slightly better on written and online versions of the AMT compared to the interview version [5].

In this task, participants completed a written version of the task where they are given two minutes to write a description of an autobiographical memory or future event in response to a cue word. A total of nine word cues were used, three of which were neutral (e.g., “house, book”), three positive (e.g., “excited”, “relaxed”) and three disorder-relevant (e.g., “stigma”, “hunger”). Participants were instructed to describe a memory, or generate a future episode, that is inspired by or related to the cue word. Aligning with the standardised instructions of the AMT and EFT-T, they were also instructed that the memory should be a specific, personal experience occurring in a time period of no longer than a day, that the future event should be a hypothetical or likely event that they would be personally involved in, that they should use a different memory/future event for each cue, and that they should consider as many details of the memory/future event as possible (i.e., what is/was happening, who they are/were with, feelings and emotions). Participants were also given two examples of responses to word cues prior to the testing trials commencing.

The word cues presented to participants were identical to those used in the previous study [21, 22], which originally were counterbalanced between participants and between tasks. In the current study, participants that previously viewed List A in the AMT task and List B in the EFT-T task, this time viewed List B in the AMT task and List A in the EFT-T task. Cues were presented in a fixed order: neutral, positive, then disorder-relevant. Participants completed the nine memory trials before the nine future event trials, with a 30 s rest break between trials.

The main dependent variable was the specificity of the response, which were coded by a trained researcher (JK) using the AMT and EFT-T manuals. Responses were coded for specificity as follows: 1 = a specific event that occurred/could occur; 2 = an extended memory/future thought, occurring over a period longer than 24 h; 3 = a categoric (repeated/repeatable) memory/future event; 4 = a semantic associate; or 5 = an off-task response. Akin to the original study [21, 22], specificity ratings were scored as an ordinal variable. On a series of 7-point Likert scales (1 = not at all, 7 = very much), participants were also asked to rate how positive, detailed, realistic, vivid and difficult to remember/imagine the memory/future event was.

### Procedure

Akin to the previous study [21, 22], all procedures took place remotely using the online platforms Gorilla (www.gorilla.sc, [1]) and Qualtrics (www.qualtrics.com). Participants that consented to being recontacted for further studies were approached via e-mail correspondence and were provided with a participant information sheet. Individuals that responded with interest were sent an informed consent form. On completion of the consent form, participants were sent the battery of questionnaires and a link to the AMT and EFT-T task online interface. The study took the form of one online testing session taking approximately one hour to complete, with optional 2-min breaks. Participants were instructed to complete the questionnaires first, followed by the online tasks (within 24 h). At the beginning of the AMT and EFT-T tasks, participants were instructed on how to optimise the study environment, such as turning their mobile phones off, making sure they were in a quiet place with few distractions, and putting the browser on full-screen. At the end of the tasks, a 3-min positive mood induction of relaxing music was played.

The study received ethical approval from the Camden and King’s Cross Research Ethics Committee (REF: 21/LO/0338). Participants were reimbursed for their time.

### Statistical analysis

Analyses were conducted using SPSS [18]. Demographic and clinical variables at TP1 were summarised descriptively. Paired-samples two-sided *t*-tests were conducted to assess changes in anthropometric and psychopathological variables from TP0 to TP1.

Changes in AMT and EFT-T outcomes from TP0 to TP1 were examined using 2 (Timepoint: baseline, follow-up) × 3 (Cue valence: positive, negative, neutral) repeated measures ANCOVAs, with age as a covariate. If significant, interaction effects were investigated with Bonferroni-adjusted post-hoc tests.

*Exploratory* Spearman’s correlations were performed to assess the association between AMT and EFT specificity at baseline and follow-up, and follow-up BMI and measures of eating disorder psychopathology, depression symptoms and identity.

## Results

### Follow-up demographic and clinical characteristics

Table 1 displays the demographic and clinical variables of the participant group at TP0 and TP1. All participants were female between the ages of 21 and 53. All participants identified as white, apart from one who identified as mixed race. At baseline, a quarter of participants were unemployed and the rest were working or studying, and most lived in their own homes (n = 14; 70%). At follow-up, most participants were employed (n = 14; 70%), and lived in their own homes (n = 15; 75%).

**Table 1:** Demographic and clinical characteristics of the AN group at TP0 and TP1 (n = 20)

Variable	AN-TP0		AN-TP1	
	M ± SD or N (%)	Range	M ± SD or N (%)	Range
Age (years)	28.4 ± 7.9	18–50	32.3 ± 8.6	21–53
Sex				
Female	20 (100%)		20 (100%)	
Male	0 (0%)		0 (0%)	
Non-binary	0 (0%)		0 (0%)	
Education (years)	16.9 ± 1.9	14–23	18.7 ± 2.5	15–26
Employment status				
Unemployed	5 (25%)		2 (10%)	
Full-time employed	7 (35%)		7 (35%)	
Part-time employed	3 (15%)		7 (35%)	
Studying	4 (20%)		4 (20%)	
Living status				
Own house (owned)	7 (35%)		10 (50%)	
Own house (rented)	7 (35%)		5 (25%)	
With parents	4 (20%)		2 (10%)	
Student accommodation	2 (10%)		3 (15%)	
Psychotropic medication usage				
Antidepressants	5 (25%)		6 (30%)	
Antipsychotics	3 (15%)		2 (10%)	
Anti-anxiety	1 (5%)		1 (5%)	
ADHD medication	0 (0%)		1 (5%)	
Comorbidities				
Depressive disorder	8 (40%)		8 (40%)	
Anxiety disorder	5 (25%)		10 (50%)	
ASD	2 (10%)		2 (10%)	
Treatment during period				
Inpatient ED treatment			4 (20%)	
Outpatient ED treatment			10 (50%)	
Day patient ED treatment			3 (15%)	
Dietician			7 (35%)	
No treatment			5 (25%)	
LEQ total score			23.1 ± 16.0	1–62
Positive events			15.4 ± 12.1	1–39
Negative events			7.7 ± 8.0	0–28
SCIM total			84.4 ± 18.9	57–121
Consolidated identity			47.8 ± 7.9	34–59
Disturbed identity			31.1 ± 9.6	12–47
Lack of identity			20.8 ± 9.3	7–40

*ADHD* attention-deficit hyperactivity disorder, *AN* anorexia nervosa, *ASD* autism spectrum disorder, *LEQ* Life Events Questionnaire, *M* mean, *N* number, *SCIM* Self-Concept and Identity Measure, *SD* standard deviation, *TP1* time-point 1

The majority reported that they were not recovered at TP1 (n = 17), although three participants reported feeling recovered despite being underweight, which they broadly defined as “eating without restriction”. Most participants had a remaining diagnosis of AN (n = 17) whilst two reported having a current diagnosis of bulimia nervosa and in one, atypical AN. Only eight (40%) of the group reported that they were currently receiving treatment.

In the time since TP0, four (20%) reported having an inpatient admission for their ED (ranging from 3 weeks to one year in length), ten (50%) reported receiving outpatient care, three (15%) reported receiving day-patient care, and seven (35%) saw a dietician at least once. Inpatient admission lengths ranged from one 3-week stay to four 6-month stays. Five (25%) received no care for their ED. At TP1, the mean ± SD time since diagnosis was 14.1 ± 8.6 years (range 3–37 years). Nineteen of 20 (95%) participants had an illness duration of > 3 years, and 14 of 20 (70%) of participants had an illness duration of > 7 years.

### Pre-post comparison of anthropometric and clinical characteristics

**Fig. 1:**
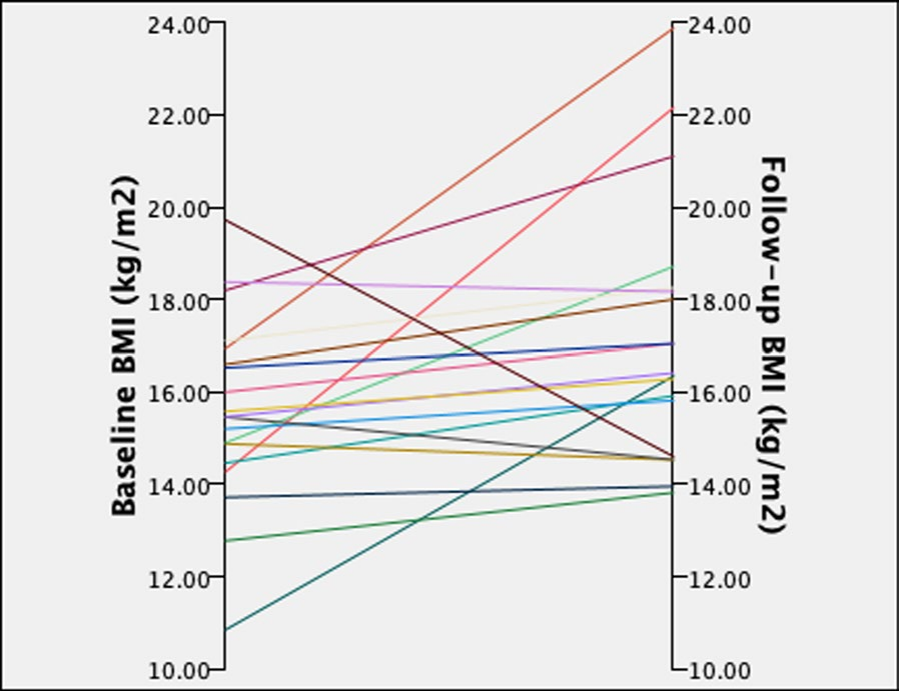
Individual participant changes in body mass index (kg/m^2^) from baseline to follow-up

BMI significantly increased from TP0 to TP1 (*p* = 0.033; Table 2), although most participants remained underweight at TP1 (n = 15, 75% under a BMI of 18.5 kg/m^2^, see Fig. 1). The mean ± SD BMI increase was 1.55 ± 2.93 kg/m^2^. Average sleep over the previous three nights was significantly lesser at follow-up (*p* = 0.040). However, there were no changes over time in ED psychopathology, sleep quality, or in measures of depression, anxiety and stress.

**Table 2:** Results of the within-subjects t-tests of clinical characteristics from TP0 to TP1

Variable	N	AN-TP0 M ± SD	AN-TP1 M ± SD	*t* (df)	95% CI	*p* value (Cohen’s *d)*
BMI, kg/m^2^	19	15.6 ± 2.1	17.2 ± 2.8	− 2.31	− 2.97, − 0.14	0.033* (− 0.53)
Average sleep duration (past 3 nights), hours	20	7.2 ± 1.7	6.5 ± 1.3	2.20	0.03, 1.30	0.040* (0.49)
Sleep quality, 0–100	20	58.0 ± 18.5	53.3 ± 20.2	1.52	− 1.76, 11.2	0.144 (0.34)
EDE-Q total score	20	3.5 ± 1.2	3.1 ± 1.6	1.54	− 0.15, 1.02	0.140 (0.35)
Restraint	20	3.5 ± 1.6	2.9 ± 1.8	1.59	− 0.20, 1.50	0.128 (0.36)
Eating concern	20	3.0 ± 1.1	2.5 ± 1.6	1.31	− 0.25, 1.06	0.205 (0.29)
Weight concern	20	3.6 ± 1.5	3.2 ± 1.9	1.03	− 0.34, 1.00	0.315 (0.23)
Shape concern	20	4.0 ± 1.2	3.7 ± 1.6	1.21	− 0.25, 0.93	0.240 (0.27)
DASS-21 depression	20	17.8 ± 12.7	17.3 ± 19.5	0.12	− 7.98, 8.98	0.903 (0.03)
DASS-21 anxiety	20	12.0 ± 8.6	11.0 ± 18.4	0.25	− 7.38, 9.38	0.805 (0.06)
DASS-21 stress	20	23.6 ± 10.9	22.6 ± 17.3	0.34	− 5.20, 7.20	0.739 (0.08)
GAD-7	20	12.5 ± 6.1	11.0 ± 5.7	1.13	− 1.27, 4.27	0.272 (0.25)

*AN* anorexia nervosa, *BMI* body mass index, *CI* confidence intervals, *df* degrees of freedom, *EDE-Q* Eating Disorder Examination-Questionnaire, *DASS-21* Depression, Anxiety and Stress Scale-21, *GAD-7* Generalised Anxiety Disorder Questionnaire, *M* mean, *n* number, *SD* standard deviation

### Comparison of AMT and EFT-T outcomes at baseline and follow-up

Table S3 details the descriptive statistics for the AM and EFT-T outcomes at baseline and follow-up. For the main AM and EFT-T outcomes (specificity) the Timepoint x Valence interaction term, and main effects of Timepoint and Valence were non-significant, indicative of no changes in AM or EFT specificity over time (Table 3). Results were the same even when accounting for changes in BMI (Table S4).

**Table 3:** Results of repeated-measures ANCOVAs on Autobiographical Memory Test and Episodic Future Thinking Task outcomes

Variable	Autobiographical memory test			Episodic future thinking task		
	F	Df	*p* value (η_p_^2^)	F	Df	*p* value (η_p_^2^)
*Specificity*						
Timepoint × valence	0.48	2, 32	0.625 (0.026)	1.19	2, 30	0.317 (0.065)
Timepoint	0.55	1, 16	0.467 (0.030)	0.19	1, 15	0.672 (0.011)
Valence	0.44	2, 32	0.647 (0.024)	1.84	2, 30	0.174 (0.098)
Age	5.24	1, 16	0.034* (0.226)	0.29	1, 15	0.595 (0.017)
*Positivity*						
Timepoint × valence	0.29	2, 32	0.747 (0.016)	2.87	2, 30	0.071 (0.211)
Timepoint	2.33	1, 16	0.144 (0.115)	1.74	1, 15	0.205 (0.093)
Valence	4.49	2, 32	0.018* (0.200)	20.35	2, 30	< 0.001** (0.494)
Age	0.43	1, 16	0.532 (0.023)	0.37	1, 15	0.552 (0.021)
*Detailedness*						
Timepoint × valence	2.02	2, 32	0.147 (0.101)	1.62	2, 30	0.214 (0.087)
Timepoint	0.01	1, 16	0.973 (0.001)	2.01	1, 15	0.174 (0.106)
Valence	2.66	2, 32	0.084 (0.129)	2.15	2, 30	0.132 (0.112)
Age	2.04	1, 16	0.170 (0.102)	0.74	1, 15	0.402 (0.042)
*Realisticness*						
Timepoint × valence	1.05	2, 32	0.362 (0.055)	1.11	2, 30	0.341 (0.061)
Timepoint	1.47	1, 16	0.242 (0.075)	0.01	1, 15	0.930 (0.001)
Valence	1.33	2, 32	0.278 (0.069)	3.75	2, 30	0.034* (0.181)
Age	0.38	1, 16	0.545 (0.021)	0.25	1, 15	0.623 (0.015)
*Vividness*						
Timepoint × valence	3.12	2, 32	0.056 (0.148)	1.05	2, 30	0.362 (0.058)
Timepoint	0.05	1, 16	0.824 (0.003)	4.41	1, 15	0.051 (0.206)
Valence	0.36	2, 32	0.702 (0.019)	2.39	2, 30	0.107 (0.123)
Age	2.94	1, 16	0.104 (0.140)	1.82	1, 15	0.196 (0.096)
Difficulty to remember/imagine						
Timepoint × valence	0.62	2, 32	0.545 (0.033)	0.88	2, 30	0.423 (0.049)
Timepoint	0.12	1, 16	0.731 (0.007)	0.06	1, 15	0.804 (0.004)
Valence	0.80	2, 32	0.457 (0.043)	2.33	2, 30	0.113 (0.120)
Age	0.10	1, 16	0.751 (0.006)	0.01	1, 15	0.919 (0.001)

**Significant at the *p* < 0.01 threshold; *significant at the *p* < 0.05 threshold

For the positivity of autobiographical memories, there was a significant main effect of cue valence whereby memories elicited by positive cues were more positive than those elicited by neutral and negative cues, and neutral more than negative (all *p* < 0.001).

Additionally, there were significant main effects of cue valence for the positivity of EFTs. As with AMs, future events elicited by positive cues were more positive than those elicited by neutral and negative cues, and neutral more than negative (all *p* < 0.001). Finally, there was a main effect of cue valence for EFT realisticness ratings, although post-hoc tests were not significant (all *p* > 0.05).

### Associations between autobiographical memory and future thinking specificity and follow-up BMI, eating disorder psychopathology, depressive symptoms and identity measures

There were broadly no significant correlations between baseline and follow-up AMT and EFT-T specificity and follow-up BMI, ED psychopathology, DASS-Depression and identity measures (Table 4). The identity domain “consolidated identity” at follow-up was moderately positively correlated with baseline AMT specificity scores.

**Table 4:** Spd follow-up AMT and EFT-T specificity and follow-up measures of body mass index, eating disorder psychopathology, depression and identity disturbance

Variable	Follow-up BMI *rho* (*p* value)	Follow-up EDE-Q global *rho* (*p* value)	Follow-up DASS-D *rho* (*p* value)	SCIM total *rho* (*p* value)	SCIM consolidated identity *rho* (*p* value)	SCIM disturbed identity *rho* (*p* value)	SCIM lack of identity *rho* (*p* value)
Baseline AMT specificity	− 0.17 (0.482)	− 0.24 (0.315)	− 0.06 (0.801)	− 0.21 (0.398)	0.47 (0.043)*	− 0.24 (0.331)	− 0.13 (0.594)
Baseline EFT-T specificity	− 0.09 (0.707)	− 0.02 (0.942)	0.28 (0.239)	0.24 (0.321)	0.01 (0.984)	0.05 (0.853)	0.22 (0.360)
FU AMT specificity	− 0.13 (0.608)	0.41 (0.071)	0.36 (0.115)	− 0.04 (0.883)	0.21 (0.389)	− 0.27 (0.261)	0.01 (0.981)
FU EFT-T specificity	− 0.31 (0.209)	0.09 (0.704)	0.43 (0.065)	0.06 (0.806)	− 0.05 (0.861)	− 0.02 (0.925)	0.03 (0.901)

*AMT* Autobiographical Memory Test, *BMI* body mass index, *DASS-D* Depression, Anxiety and Stress Scale-Depression, *EDE-Q* Eating Disorder Examination-Questionnaire, *EFT-T* Episodic Future Thinking Task, *FU* follow-up, *SCIM* Self-Concept and Identity Measure

*Significant at the *p* < 0.05 threshold

## Discussion

This study aimed to examine the stability of AM and EFT performance over time, and to examine associations between baseline OGM (AM specificity) and follow-up ED-related and identity outcomes. In this sample of respondents (approximately 50% of the original sample), there was little evidence of overall eating disorder recovery, as ED psychopathology and depression scores remained high at follow-up. BMI had significantly increased over the three years and had increased in 75% of the sample, although the average BMI at follow-up was still low (17.2 kg/m^2^). As the respondents were largely unrecovered, our study was unable to interrogate the prognostic value of baseline OGM in terms of long-term outcomes. Indeed in this sample, correlation analyses indicated that AM and EFT specificity scores both at baseline and follow-up were not associated with BMI, eating disorder psychopathology, depression symptoms or measures of identity disturbance and self-concept.

When examining AM and EFT performance longitudinally, the main outcome of specificity was unchanged over the course of the three years, aligning with our expectation that the ability to generate specific AMs would remain poor if participants were unrecovered [21, 22]. These findings remained consistent even when accounting for changes in weight over time. Contrasting with findings from Terhoeven et al. [39], AM performance remained poor for memories regardless of the type of memory cue, although the BMI increase in our sample was considerably smaller (1.6 kg/m^2^ versus 3.1 kg/m^2^). It is likely that a lack of statistical power contributed to our results. According to the measure of effect size, there were moderate-sized decreases in the participant ratings of how positive and realistic recalled AMs were, as well as moderate-sized and large-sized decreases in participant ratings of the positivity and vividness of EFTs, respectively. This may be a preliminary indicator of that interpretations of memories as well as projections to the future may increase in negativity over time in people with AN who remain unrecovered.

The sample participating in this follow-up had persistently high ED psychopathology and symptoms of depression, anxiety and stress, and although BMI increased, the average BMI still remained low. The original sample from the initial study had a long illness duration of an average of 8.7 years [21, 22], and it is possible that this could have contributed to the low incidence of recovery in the respondents. Indeed, the follow-up sample would perhaps be best characterised as mostly longstanding AN (or “severe-enduring” AN), as most had an illness duration of over 7 years. There were no observable differences in baseline characteristics between those who responded to the advertisement and those who did not, although it is conceivable that individuals who had recovered may be less inclined to participate in research. The present study was unable to explore the prognostic value of AM performance in terms of recovery or remission in AN, and a well-controlled prospective longitudinal study assessing individuals over the course of weight restoration would be beneficial to examine this further—for example whether better baseline AM performance is related to better outcomes over time. It is unclear whether the correlational results observed in this study would generalise to samples where follow-up outcomes were more variable (i.e., where some had recovered).

In this sample, AM and EFT performance remained stable over time despite a slight increase in weight. It is presumable that with this increase in weight, some level of volumetric recovery in the brain occurred [34], although problems with AM persisted. This may indicate that full weight (and/or symptomatic) recovery is necessary for the reversal of AM deficiencies. Prior cross-sectional data indicates that OGM is not apparent in people who are long-term recovered from AN, pointing towards reversal of OGM following long-term recovery [21, 22]. Other studies examining changes in AM specificity after short-term weight restoration treatment have conflicting findings [38, 39]. The temporal dynamics of AM abilities over the course of recovery are unclear, as are the mechanisms involved in these changes. For example, it is possible that changes in AM over the course of recovery may be related to changes in biology (e.g., brain volume recovery), changes in allied cognitive domains, and/or changes in ED symptoms, or in symptoms of psychiatric comorbidity such as depression. Indeed, in our sample both ED psychopathology and depression scores remained high at follow-up, which could contribute to persisting OGM. However, in both the original study [21, 22] and this follow-up study, symptoms of depression were not associated with AM specificity. Moreover, in the original study, AM and EFT performance was intact in people recovered from AN despite them still having higher depression scores in comparison to controls. Therefore, it is unlikely that persisting symptoms of depression are the sole contributor to the OGM observed in AN.

Given that processes of AM have been linked to wider processes of identity consolidation and development, we hypothesised that AM specificity would be negatively related to identity and self-concept disturbances, although this was not found. Conway’s “Self Memory System” [7] describes a tripartite structure that links memory to the self, made up of the conceptual self (semantic representations of one’s personal characteristics), autobiographical knowledge (information on the structure of one’s life) and episodic memory (sensory, contextual, cognitive and emotional details of specific experiences). These three aspects can be selectively impaired [14, 23, 31] and may be mediated by distinct neural signatures [26]. Self-conceptions are dynamic and can be modulated by autobiographical retrieval, and certain autobiographical memories can represent “self-defining memories”, which play a more central role in forming one’s overarching self-concept [35]. We did not purposefully instruct participants to generate self-defining memories, which may explain the lack of association with measures of identity disturbance. Future studies should endeavour to interrogate the “Self Memory System” in people with AN, perhaps through experimental studies examining whether AM retrieval or EFT generation modifies self-concept [10].

### Strengths and limitations

There are several limitations to the present study. The first and most important limitation is the small number of respondents, which both reduces statistical power and also resulted in a low variance in terms of outcome, meaning that we were unable to interrogate all prespecified hypotheses in detail. Therefore, these hypotheses in question, namely the prognostic value of AM deficiencies, should be interrogated in a future prospective longitudinal study with a larger sample that is more diverse in terms of outcome. Relatedly, no in-depth clinical interview was performed to verify the presence or absence of an AN diagnosis, and all variables were self-reported meaning that some outcomes (e.g., weight) could not be verified. However, it has been found that self-reported weight has good accuracy in people with AN [27, 28] and the same procedure was used for the baseline assessment [21, 22]. Similarly, the task was remotely administered so as to utilise the same protocol as the baseline assessment and limit noise in the data, which does not reduce the validity of the results per se but should be considered when comparing the findings to other studies that administered the AMT and EFT-T in-person. Importantly, this sample may be characterised as having longstanding AN, and were mostly highly educated, all female and white, with many owning their own home. Therefore, it is apparent that although this group was homogenous in terms of demographics, this limits the generalisability of the findings to people with a shorter illness duration, adolescents, non-females, other ethnicities and people with different socioeconomic backgrounds.

### Conclusions

This study examined the temporal stability of overgeneral memory (poor autobiographical specificity) in a community-based sample of 20 people with mainly longstanding AN examined after three years. The respondents were largely unrecovered in terms of ED and wider psychopathology, and despite a slight increase in weight in some, the specificity of recalled autobiographical memories and generated future events was unchanged over time. Baseline overgeneral memory was not associated with BMI or ED and depression symptoms at follow-up, however the prognostic value of autobiographical memory specificity could not be fully interrogated in this study due to the lack of variation in outcomes at follow-up. Future research would benefit from exploring the prognostic value of AM performance on ED-related outcomes and also the treatment itself in a larger clinical sample receiving care who are more likely to vary in outcome longitudinally.

## Supplementary Information

Additional file 1.

## Data Availability

No datasets were generated or analysed during the current study.
