# Supplementary material for: Autobiographical memory and episodic future thinking in anorexia nervosa: a three-year follow-up
Source: J Eat Disord. 2025 May 30;13:94. doi: 10.1186/s40337-025-01280-4 (PMC12123733; doi:10.1186/s40337-025-01280-4)
Supplement: Supplementary file 1 — Additional file 1. [file 40337_2025_1280_MOESM1_ESM.docx]

**Supplementary material for Keeler et al. Autobiographical memory and episodic future thinking in anorexia nervosa: a three-year follow-up**

**Table S1.** Comparison of baseline characteristics across responders (n=20) and non-responders (n=27) to follow-up invitation

| **Variable** | **Did not respond** | | **Responded** | |  |  |
| --- | --- | --- | --- | --- | --- | --- |
|  | **N** | **M±SD or %** | **N** | **M±SD or %** | ***t* or *X*^2^** | ***p*-value** |
| Age, years | 27 | 26.63 ± 6.62 | 20 | 28.4 ± 7.88 | -0.84 | 0.408 |
| Sex |  |  |  |  |  |  |
| Female | 24 | 89% | 20 | 100% | 2.37 | 0.123 |
| Non-binary | 3 | 11% | 0 | 0% |  |  |
| Male | 0 | 0% | 0 | 0% |  |  |
| Ethnicity |  |  |  |  |  |  |
| White | 25 | 93% | 19 | 95% | 0.11 | 0.739 |
| Mixed race | 2 | 7% | 1 | 5% |  |  |
| Education (years) | 27 | 16.41 ± 2.41 | 20 | 16.9 ± 1.92 | -0.76 | 0.454 |
| AN subtype |  |  |  |  |  |  |
| AN-R | 17 | 63% | 15 | 75% | 3.81 | 0.149 |
| AN-BP | 3 | 11% | 4 | 57% |  |  |
| BMI, kg/m^2^ | 27 | 16.71 ± 2.62 | 20 | 15.62 ± 2.13 | 1.53 | 0.133 |
| Lowest BMI, kg/m^2^ | 27 | 13.81 ± 2.12 | 20 | 13.49 ± 2.35 | 0.48 | 0.637 |
| Illness duration, years | 27 | 11.48 ± 5.32 | 20 | 13.82 ± 8.36 | -1.17 | 0.248 |
| EDE-Q Total score | 27 | 3.64 ± 1.35 | 20 | 3.57 ± 1.16 | 0.18 | 0.855 |
| Restraint | 27 | 3.60 ± 1.81 | 20 | 3.64 ± 1.45 | -0.08 | 0.936 |
| Eating concern | 27 | 3.45 ± 1.57 | 20 | 2.94 ± 1.14 | 1.24 | 0.223 |
| Weight concern | 27 | 3.41 ± 1.48 | 20 | 3.59 ± 1.45 | -0.40 | 0.688 |
| Shape concern | 27 | 4.10 ± 1.31 | 20 | 4.11 ± 1.14 | -0.05 | 0.959 |
| DASS-21 Depression | 27 | 20. 37 ± 12.47 | 20 | 17.30 ± 12.47 | 0.84 | 0.408 |
| DASS-21 Anxiety | 27 | 15.19 ± 9.88 | 20 | 12.20 ± 8.63 | 1.08 | 0.286 |
| DASS-21 Stress | 27 | 22.96 ± 9.57 | 20 | 23.90 ± 10.69 | -0.32 | 0.754 |
| GAD-7 | 27 | 12.63 ± 4.90 | 20 | 12.65 ± 6.12 | -0.01 | 0.990 |

Abbreviations. AN = anorexia nervosa; AN-BP = AN binge-purge subtype; AN-R = AN restricting subtype; BMI = body mass index; EDE-Q = Eating Disorder Examination-Questionnaire; DASS-21 = Depression, Anxiety and Stress Scale-21; GAD-7 = Generalised Anxiety Disorder Questionnaire; M = mean; n = number; SD = standard deviation.

**Table S2.** Test-retest reliability estimates from baseline to 3-year follow-up using Spearman’s correlation coefficients

| **Variable** | **Spearman’s rho** | ***p*-value** |
| --- | --- | --- |
| AMT |  |  |
| Specificity | .18 | .453 |
| Positivity | .24 | .301 |
| Detailedness | .40 | .084 |
| Realisticness | .25 | .287 |
| Vividness | .33 | .162 |
| Difficulty to remember | .10 | .680 |
| EFT-T |  |  |
| Specificity | .18 | .471 |
| Positivity | -.39 | .104 |
| Detailedness | .38 | .106 |
| Realisticness | .16 | .509 |
| Vividness | .37 | .116 |
| Difficulty to imagine | .40 | .090 |
| EDE-Q | .60 | .005** |
| Global |  |  |
| Restraint | .47 | .036* |
| Eating concern | .50 | .024* |
| Weight concern | .61 | .004** |
| Shape concern | .62 | .003** |
| DASS-Depression | .51 | .021* |
| DASS-Anxiety | .38 | .095 |
| DASS-Stress | .74 | .001** |
| GAD-7 | .48 | .031* |

**Significant at the *p*<0.001 threshold, *Significant at the *p*<0.05 threshold. Abbreviations: AMT = Autobiographical Memory Test; DASS = Depression, Anxiety and Stress Scale; EDE-Q = Eating Disorder Examination-Questionnaire; EFT-T = Episodic Future Thinking-Test; GAD-7 = Generalised Anxiety Disorder-7.

**Table S3.** Descriptive statistics for AMT and EFT-T outcomes at baseline and follow-up

| **Task** | **Variable** | **Baseline**  **M±SD** | | | | **Follow-up**  **M±SD** | | | |
| --- | --- | --- | --- | --- | --- | --- | --- | --- | --- |
|  |  | **Overall** | **Positive** | **Neutral** | **Negative** | **Overall** | **Positive** | **Neutral** | **Negative** |
| **AMT** | Specificity | 1.71 ± 0.64 | 1.95 ± 1.13 | 1.47 ± 0.61 | 1.70 ± 0.67 | 1.97 ± 0.79 | 2.15 ± 1.06 | 1.83 ± 0.83 | 1.93 ± 0.94 |
|  | Positivity | 3.67 ± 0.56 | 5.02 ± 1.17 | 3.88 ± 1.06 | 2.12 ± 0.60 | 3.62 ± 0.84 | 5.02 ± 1.35 | 3.92 ± 1.43 | 1.92 ± 0.88 |
|  | Detailedness | 4.61 ± 1.08 | 4.40 ± 1.31 | 5.05 ± 1.10 | 4.38 ± 1.34 | 4.45 ± 1.22 | 4.40 ± 1.40 | 4.83 ± 1.37 | 4.12 ± 1.45 |
|  | Realisticness | 5.56 ± 0.91 | 5.47 ± 1.06 | 5.87 ± 0.98 | 5.33 ± 1.10 | 5.47 ± 0.98 | 5.32 ± 1.08 | 5.72 ± 1.23 | 5.38 ± 1.23 |
|  | Vividness | 4.85 ± 1.01 | 4.42 ± 1.35 | 5.28 ± 1.01 | 4.85 ± 1.24 | 4.92 ± 1.16 | 4.67 ± 1.48 | 5.27 ± 1.19 | 4.82 ± 1.28 |
|  | Difficulty to remember | 2.64 ± 0.72 | 2.92 ± 1.11 | 2.35 ± 0.93 | 2.65 ± 1.20 | 2.89 ± 1.15 | 3.13 ± 1.51 | 2.32 ± 1.09 | 3.23 ± 1.90 |
| **EFT-T** | Specificity | 1.97 ± 0.74 | 1.87 ± 0.93 | 1.69 ± 0.75 | 2.35 ± 1.14 | 1.98 ± 0.73 | 1.79 ± 0.61 | 1.82 ± 0.76 | 2.32 ± 1.23 |
|  | Positivity | 4.20 ± 0.84 | 5.40 ± 1.21 | 4.83 ± 1.25 | 2.37 ± 0.91 | 4.15 ± 0.74 | 5.68 ± 1.11 | 4.56 ± 1.24 | 2.19 ± 0.85 |
|  | Detailedness | 4.11 ± 0.94 | 4.27 ± 1.12 | 4.32 ± 1.24 | 3.75 ± 1.08 | 4.19 ± 0.97 | 4.53 ± 1.15 | 4.51 ± 1.26 | 3.54 ± 1.22 |
|  | Realisticness | 4.73 ± 0.76 | 4.73 ± 0.98 | 4.80 ± 1.17 | 4.65 ± 1.23 | 4.79 ± 0.80 | 5.07 ± 1.19 | 5.04 ± 1.04 | 4.26 ± 1.39 |
|  | Vividness | 4.21 ± 0.89 | 4.15 ± 1.13 | 4.32 ± 1.08 | 4.15 ± 0.93 | 4.16 ± 1.25 | 4.42 ± 1.50 | 4.47 ± 1.52 | 3.60 ± 1.29 |
|  | Difficulty to remember | 3.32 ± 0.91 | 3.32 ± 1.38 | 3.30 ± 1.26 | 3.33 ± 1.38 | 3.22 ± 1.17 | 2.98 ± 1.45 | 2.86 ± 1.42 | 3.82 ± 1.36 |

Abbreviations. AMT = Autobiographical Memory Test; EFT-T = Episodic Future Thinking Task; M = mean; SD = standard deviation.

**Table S4.** Results of repeated-measures ANCOVAs on Autobiographical Memory Test and Episodic Future Thinking Task outcomes, controlling for changes in BMI (ΔBMI)

| **Variable** | **Autobiographical Memory Test** | | | **Episodic Future Thinking Task** | | |
| --- | --- | --- | --- | --- | --- | --- |
|  | **F** | **Df** | **p-value (η_p_^2^)** | **F** | **Df** | **p-value (η_p_^2^)** |
| **Specificity** | | | |  |  |  |
| Timepoint x Valence | 0.80 | 2 | 0.457 (0.048) | 0.97 | 2 | 0.390 (0.061) |
| Timepoint | 0.71 | 1 | 0.411 (0.043) | 0.02 | 1 | 0.900 (0.001) |
| Valence | 0.15 | 2 | 0.865 (0.009) | 0.54 | 2 | 0.589 (0.035) |
| Age | 3.71 | 1 | 0.072 (0.188) | 0.71 | 1 | 0.411 (0.045) |
| ΔBMI | 0.54 | 1 | 0.474 (0.032) | 0.01 | 1 | 0.916 (0.001) |

Abbreviations. BMI = body mass index; η_p_^2^ = partial eta squared.
